# C57BL/6 Substrain Differences in Pharmacological Effects after Acute and Repeated Nicotine Administration

**DOI:** 10.3390/brainsci9100244

**Published:** 2019-09-21

**Authors:** Lois S. Akinola, Bryan Mckiver, Wisam Toma, Andy Z. X. Zhu, Rachel F. Tyndale, Vivek Kumar, M. Imad Damaj

**Affiliations:** 1 Department of Pharmacology and Toxicology, and Translational Research Initiative for Pain and Neuropathy, Medical College of Virginia Campus, Virginia Commonwealth University, Richmond, VA 23298, USA; akinolals@vcu.edu (L.S.A.); mckiverbd@vcu.edu (B.M.); tomawb@vcu.edu (W.T.); 2 Takeda Pharmaceutical Company Limited, Cambridge, MA 02139, USA; zhuzix@gmail.com; 3 Campbell Family Mental Health Research Institute, Centre for Addiction and Mental Health (CAMH), Department of Pharmacology and Toxicology, and Psychiatry, University of Toronto, Toronto, ON, Canada; r.tyndale@utoronto.ca; 4 The Jackson Laboratory, Bar Harbor, ME 04609, USA; vivek.kumar@jax.org

**Keywords:** nicotine, acute effects, mice, C57BL/6 substrains

## Abstract

Tobacco smoking is the major cause of disability and death in the United States and around the world. In addition, tobacco dependence and addiction express themselves as complex behaviors involving an interplay of genetics, environment, and psychological state. Mouse genetic studies could potentially elucidate the novel genes and/or gene networks regulating various aspects of nicotine dependence. Using the closely related C57BL/6 (B6) mice substrains, recent reports have noted phenotypic differences within C57BL/6J (B6J) and C57BL/6N (B6N) mice for some drugs of abuse: alcohol, opiates, and cocaine. However, the differences in nicotine’s effects have not yet been described in these substrains. We examined the phenotypic differences in these substrains following the acute and repeated administration of nicotine in several pharmacological measures, including locomotion (after acute and repeated exposure), body temperature, nociception, and anxiety-like behaviors. We report substrain differences in the pharmacological effects of acute and repeated nicotine administration in the B6 substrains. Overall, we show enhanced nicotine sensitivity to locomotion, hypothermia, antinociception, and anxiety-like behaviors in the B6J mouse substrain compared to B6N. In the repeated administration paradigm, both the B6N and B6J substrains showed no sensitized locomotor responses after repeated exposure to nicotine at the two doses tested. This study thus provides evidence that the B6 mouse substrains may be useful for genetic studies to elucidate some of the genetic variants involved in tobacco dependence and addiction.

## 1. Introduction

The World Health Organization (WHO) reports more than seven million deaths each year from direct tobacco use, which is estimated to rise to 10 million deaths per year by the year 2030 [1,2,3]. Tobacco smoking, however, is the major cause of disability and death in the United States and around the world [2]. Many smokers still face immense difficulty quitting and remaining tobacco-free permanently despite numerous informational efforts on the hazards of tobacco smoking and other control efforts [4,5,6]. Tobacco dependence expresses as a complex interplay of several behaviors, likely involving an even more complex play of several genes, each making a small contribution. Twin studies reported a tobacco dependence heritability estimate of 50–70% [7,8]. Over the years, several nicotinic receptor gene variants have been implicated in nicotine dependence (*CHRNA5-CHRNA3-CHRNB4* within the q25.1 region of chromosome 15, as well as in the primary metabolism gene, *CYP2A6*). However, these genetic variants explain only a small proportion of the genetic risk of tobacco-use initiation and dependence [9,10,11]. The successful identification of replicable “nicotine addiction genes” has eluded the scientific community for many decades, owing to the lack of consistent definitions of phenotypes across various cohorts, as well as a deficit in statistical power [12]. A discovery-based approach to genetic mapping known as “Quantitative trait locus (QTL) mapping” has been successfully used in the identification of genetic variants underlying several complex phenotypes [13,14]. This approach allows the mapping of regions of polymorphism within the genome to narrow intervals, thus facilitating the identification of causative variants associated with complex behaviors. However, while preclinical QTL mapping models of dependence and addiction-like behaviors have been described in numerous rodent studies with alcohol and other drugs of abuse, very few reports exist for nicotine. Significant QTLs in mice using F2 crosses were identified for oral nicotine intake in the preference test [15] and in the effect of systemic nicotine on locomotor activity [16,17]. However, no confirmation or fine-mapping for potential candidate genes were reported in these studies, which is probably due to the high genetic complexity underlying a QTL, combined with large linkage disequilibrium blocks which can hinder gene identification [14]. 

Reduced complexity crosses (RCC) offer a solution and have been successfully used to identify quantitative trait nucleotides (QTN). Compared to the inbred reference strain, C57BL/6J (BJ), most classical inbred strains contain approximately 5 million variants; the C57BL/6NJ (B6N) substrain, a closely related inbred substrain, contains roughly 30,000 variants in comparison [18,19]. Thus, between both substrains, the identification of causal genes and variants using QTL mapping is greatly facilitated due to the stark reduction in genetic complexity [20,21,22,23]. In our previous report, we mapped the genetic basis of variation in cocaine’s effects on locomotor activation and sensitization within parental strains using the C57BL/6 (B6) substrains [22]. We report substrain differences in B6N versus B6J mice, where the B6N substrain demonstrated a lower response to both cocaine and methamphetamine in an acute and sensitized model compared to B6J mice. Using QTL mapping, we identified a causative variant on QTL mid-chromosome 11 that produced a nonsynonymous mutation (S968F) in the cytoplasmic FMRP interacting protein 2 (Cyfip2). These results provided support for Cyfip2 as a causal agent underlying cocaine’s effects on locomotor activity and sensitivity. This same variant was identified in another QTL mapping study for binge eating behavior [23]. Thus, Cyfip2 seems to regulate both natural and drug-induced behaviors. 

The emergence of tobacco dependence is primarily due to the psychoactive properties of nicotine. Nicotine has broad pharmacological effects on humans and animals [24]. Under first exposure to nicotine, either by inhalation or through other routes of administration, subjects report relaxation, alertness, dizziness, and nausea, amongst other effects [25]. These initial responses may play significant roles in sustained smoking and the subsequent development of dependence to nicotine. However, in most human genetic studies, these initial effects are rarely included in association testing. Importantly, early animal behavioral studies established that various inbred mice strains were differentially sensitive to the initial effects of nicotine. These genetic analyses showed that nicotine’s pharmacological effects on locomotion, body temperature, nociception, and seizures are heritable [26,27]. It is unknown whether nicotine’s differential pharmacological sensitivity extends to the B6 substrains. This is important to investigate before eQTLs can be performed. We, therefore, evaluated possible differences in nicotine pharmacological responses and pharmacokinetics after acute and repeated administration. Differences in nicotine’s initial responses between the B6N and B6J mouse substrains in several pharmacological measures were evaluated: locomotion (after acute and repeated exposure), body temperature, nociception, and anxiety-like behaviors.

## 2. Materials and Methods

### 2.1. Mice

Adult male C57BL/6J (B6J) (The Jackson Laboratory; JAX—Bar Harbor, ME, USA), and C57BL/6NCrl mice (B6N) (Charles River Laboratories—Wilmington, MA, USA) aged 8–10 weeks at the beginning of the experiments were used throughout the study. Mice were acclimated to the vivarium for a week and handled prior to any behavioral testing. Separate cohorts of mice were used for the studies, except for their body temperatures and acute locomotor activity to avoid repetitive testing; and because nicotine has different potencies in various behavioral tests. Mice were housed in accordance to the Association for Assessment and Accreditation of Laboratory Animal Care at 21 °C in an approved animal care facility at Virginia Commonwealth University with humidity-control. Each cage was housed with groups of four mice who had free access to standard rodent chow (#7012, Envigo Teklad, Madison, WI, USA) and water throughout the study. Teklad corn cob bedding (#7097, Envigo Teklad, Madison, WI, USA) was used in the mouse cages and was changed weekly. A 12 h light/dark cycle paradigm was used, and all tests were performed within the light cycle from 7:00 to 19:00. All studies were authorized by the Institutional Animal Care and Use Committee at Virginia Commonwealth University and implemented according to the National Institutes of Health’s Guide for the Care and Use of Laboratory Animals.

### 2.2. Drugs

(-)-Nicotine hydrogen tartrate [(-)-1-methyl-2-(3- pyridyl) pyrrolidine (þ)-bitartrate] was procured from Sigma-Aldrich Inc. (St. Louis, MO, USA). Solutions were made by dissolving nicotine in physiologic saline consisting of 0.9% sodium chloride. The route of administration was through subcutaneous (s.c.) injection. Each animal received a total volume of 1 mL per 100 g (Volume/Body Weight) unless stated otherwise. Every dose is represented as the free base of the drug.

### 2.3. Behavioral Tests

#### 2.3.1. Tail-Flick Test

To test antinociception in mice, the tail-flick assay was used according to D’Amour and Smith (1941), as adapted by Dewey et al. (1970) [28,29]. Baseline measurements were obtained for all mice before treatment. Then, test-latencies were measured 5 mins after administration of either vehicle or nicotine (s.c.). To prevent damage of mouse tissue, a cut-off time latency of 10 s was set. To calculate antinociceptive response, data were computed as the percent maximum possible effect (% MPE), calculated as [(test-control)/(10-control)] × 100%.

#### 2.3.2. Hot Plate Test

The hot plate test is another test of antinociception and was performed according to the protocols reported by Eddy and Leimbach (1953), along with Atwell and Jacobson (1978) [30,31]. Baseline measurements were obtained for all mice before treatment. Then, test-latencies were measured 5 min after the administration of either the vehicle or nicotine (s.c.). Mice were observed to jump or lick their paws as a positive response. Test latencies were determined by placing mice unto a 10 cm wide glass cylinder on a hot plate (Thermojust Apparatus, Columbus, OH, USA), adjusted to 55.0 °C. A cut-off time latency of 40 s was set to prevent damage of mouse tissue. To calculate antinociceptive response, data were computed as the percent maximum possible effect (% MPE), calculated as [(test-control)/(40-control)] × 100%.

#### 2.3.3. Locomotor Activity

##### Acute Nicotine

To test for locomotion, mice were individually stationed into photocell activity cages (Omnitech Electronics, Columbus, OH, USA) measuring 28 × 16.5 cm. Photocell beam interruptions (two banks of eight cells each) were recorded for 30 min following a 5 min pretreatment of either vehicle or nicotine (0.5 or 1 mg/kg) s.c.

##### Repeated Administration 

Initially, mice injections started with subcutaneous administration of saline for three days, followed by administration of nicotine at 0.5 or 1.0 mg/kg for five days. Then, after a 2-day washout period (day 6 to day 8), mice were challenged with the same dose of nicotine. Mice were tested daily for locomotion (a similar procedure using locomotor activity boxes was followed as described above in the acute nicotine experiment) for five minutes following subcutaneous administration of either saline (days 1–3) or nicotine (days 4–8 and 12).

#### 2.3.4. Body Temperature

Body temperatures were measured rectally using a thermistor probe (inserted 24 mm) and digital thermometer (Yellow Springs Instrument Co., Yellow Springs, OH, USA). Baseline measurements were obtained prior to saline or nicotine injection and after 30 min of the injection of the same treatments. Data were calculated as the difference in rectal temperature before and after treatment. The variation of ambient temperature in the laboratory ranged from 21 °C to 24 °C.

#### 2.3.5. Elevated Plus-Maze

An elevated plus maze (EPM) was used to assess anxiety-like behaviors for this study. The EPM is made of gray plexiglass mounted on a base that is raised a total length of 60 cm from the ground. It has two open arms (23 × 6.0 cm) and two enclosed arms (23 × 6 × 15 cm), which extend from a central platform. The apparatus is illuminated by a single source of fluorescent lights (350 lux intensity) hung from the ceiling of the room. Mice were tested for 5 min following subcutaneous injection of either saline or nicotine where they are put in the center of the maze and permitted to willingly explore. The total amount of time spent within the open arms of the EPM, and the total number of crossings between the arms with the 5 min were scored automatically using a photocell beams system. After each test, the equipment was thoroughly wiped.

### 2.4. Nicotine and Cotinine Plasma Levels

Blood samples from adult male B6J and B6N mice were collected at 5, 15, 30, 60, and 90 min after subcutaneous injection of 1 mg/kg nicotine. CO_2_ was used in the anesthetization of the mice, and right before death, blood samples were collected via intra-cardiac puncture. The collected blood samples were preserved in sodium heparin blood collection tubes and subsequently centrifuged at 1400× *g* for ten min. The resulting serum was collected and then stored at −4 °C. HPLC/MS/MS analysis was used to quantify the serum levels of nicotine and cotinine in blood samples. Separate groups of mice were used.

#### 2.4.1. HPLC/MS/MS Analysis of Nicotine and Cotinine

##### Specimen Extraction

The methods for the specimen extractions were performed according to AlSharari et al., 2013 [32]. A 50 µL solution of internal standard containing 50 ng of cotinine-d3 and nicotine-d4 in methanol was added to 200 µL of aliquoted serum extracted from specimens. Next, 100 µL of ammonium hydroxide (5M), followed by 2 mL methylene chloride, was added to the mixture. The solution was vortexed and subsequently centrifuged at 3000 rpm for 5 min at 4 °C. The organic layer was extracted, and the aqueous phase was washed twice using 2 mL of methylene chloride. The resulting organic layers were then combined in a new test tube with 500 µL of HCL (25 mM) in methanol and dried using nitrogen. The samples were placed in HPLC/MS/MS auto-sampler vials for analysis after reconstituting with 100 µL of the mobile phase.

##### Instrumental Analysis

Instrumental analyses were performed using an Applied Biosystems (3200 Qtrap) and Shimadzu SCL HPLC system controlled by Analyst 1.4.2 software with turbo V source for TurbolonSpray. A 3 mm by 50 mm, 5 micron Hypersil Gold system (Thermo Scientific, Waltham, MA, USA) was used for chromatographic separation. The mobile phase, maintained at a flow rate of 0.5 mL/min, consisted of 10 mM ammonium folate and methanol at a ratio of 10:90 (V/V). A positive multiple reaction monitoring (MRM) acquisition mode was utilized; with monitored ion transitions as follows: nicotine (163 > 130; 163 > 117), nicotine-d4 (167 > 134), cotinine (177 > 80; 177 > 98), and cotinine-d3 (180 > 80). Time set for each chromatographic separation was 2 min. Based on a linear regression generated from peak area ratios of each drug to its deuterated internal standard, a calibration curve (12.5 ng/mL –500 ng/mL) was designed for each compound. The internal standard for 3-hydroxycotinine was Cotinine-d3. 

#### 2.4.2. Pharmacokinetic Analysis

A Phoenix WINNONLIN 6.4 program (Certara, Princeton, NJ, USA) was used for all pharmacokinetic calculations. Statistical comparisons of the pharmacokinetic parameters were performed using two-tailed *t*-tests. Terminal half-live values of nicotine and cotinine were obtained through extrapolation of regression analyses calculated from terminal sampling points. Each regression analysis used data from no less than three different time-points in the terminal phase, and visual inspection of each analysis was congruent with a straight line on a log-transformed scale. The trapezoidal rule was used to determine AUC_0-60_, AUC_0-90_ and AUC_0-inf_ values.

### 2.5. Statistical Analysis

Behavioral data were analyzed using GraphPad Prism software, version 8.1.2 (GraphPad Software, Inc., La Jolla, CA, USA) and expressed as the mean ± S.E.M. Statistical analyses were conducted using a two-way analysis of variance test (ANOVA), followed by a Holm-Sidak post hoc test, unless specified otherwise. ED50 values with 95% confidence limits (CL) were also calculated for the acute studies by unweighted least-squares linear regression as described by Tallarida and Murray (1987) [33]. If the confidence limit values did not overlap, the shift in the dose-response curve was considered significant. Differences were considered significant at *p* < 0.05.

## 3. Results

### 3.1. Effects of Acute Nicotine on Thermal Antinociception, Locomotion, and Body Temperature

**Figure 1:**
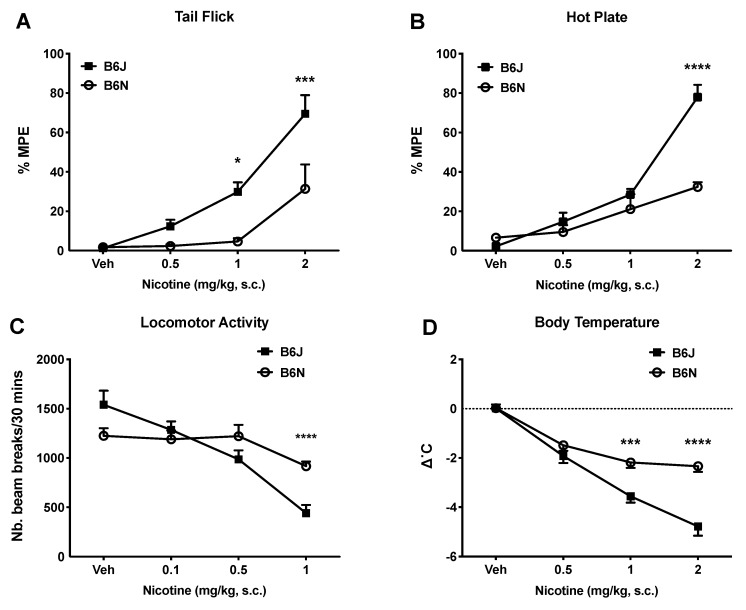
Effects of acute nicotine on thermal antinociception, locomotor activity, and body temperature in the B6J and B6N substrains. Thermal antinociception is expressed as the percentage of the maximal possible effect in (**a**) tail flick and (**b**) hot plate test. (**c**) Locomotion response to acute nicotine in male B6J and B6N mice. (**d**) Body temperature in response to acute nicotine in male B6J and B6N mice. Nicotine (0.1, 0.5, 1, and 2 mg/kg) or the vehicle (0.9% saline) was administered subcutaneously. Each data point for thermal antinociception is represented by the mean %MPE + SEM. Significant difference is based on Holm-Sidak post-hoc test following the two-way ANOVA between both substrains (* *p* < 0.05, *** *p* < 0.001, **** *p* < 0.0001), *n* = 8/group.

We investigated the effects of acute nicotine administration on thermal antinociception, locomotor activity, and body temperature. Nicotine produced a dose-responsive increase in the tail-flick latency (Figure 1A) in the B6J and B6N substrains with an ED50 (±CL) of 1.3 (0.56–1.89) and 5.9 (3.1–11.23) mg/kg, respectively (Table 1). 

A two-way ANOVA revealed significant effects of dose, strain, and interaction in the tail-flick test (F_strain_ (1, 56) = 18.73, *p* < 0.0001; F_dose_ (3, 56) = 26.95, *p* < 0.0001; F_strain×dose_ (3, 56) = 4.061, *p* = 0.0111; Figure 1A), and hot plate test (F_strain_ (1, 56) = 19.57, *p* < 0.0001; F_dose_ (3, 56) = 13.10, *p* < 0.0001; F_strain×dose_ (3, 56) = 53.84, *p* < 0.0001; Figure 1B). Similarly, the B6J substrain demonstrated an increased nicotine potency in the hot plate test in a dose-dependent manner (as shown in Figure 1B), compared to the B6N substrain with an ED50 (±CL) of 1.08 (0.83–1.38) and 7.79 (3.56–13.41) mg/kg, respectively (Table 1). While nicotine produced a dose-responsive decrease in locomotor activity in B6J mice (Figure 1C), the decrease was only significant at the dose of 1 mg/kg in the B6N substrain. The two-way ANOVA analysis revealed a significant interaction between the strain and dose on locomotor activity (F_strain×dose_ (3, 56) = 6.822, *p* = 0.0005; Figure 1C), as well as a dose-related effect of nicotine treatment (F_dose_ (3, 56) = 20.44, *p* < 0.0001; Figure 1C). Although there was no main effect of the strain in the analysis, a Holm–Sidak post hoc comparison test revealed differences in the B6J and B6N strains at the highest dose of nicotine (1.0 mg/kg) we tested. Finally, nicotine-induced hypothermia was dose-dependent in both the B6J and B6N substrains (Figure 1D) with an ED50 (±CL) of 0.57 (0.37–0.88) and 1.15 (0.96–2.13) mg/kg, respectively (Table 1). The two-way ANOVA revealed significant effects of the dose, strain, and interaction within the body temperature changes (F_strain_ (1, 56) = 38.83, *p* < 0.0001; F_dose_ (3, 56) = 83.50, *p* < 0.0001; F_strain×dose_ (3, 56) = 9.987, *p* < 0.0001; Figure 1D).

### 3.2. Dichotomous Effect of Nicotine on Anxiety-Like Behavior in the EPM Assay

**Figure 2:**
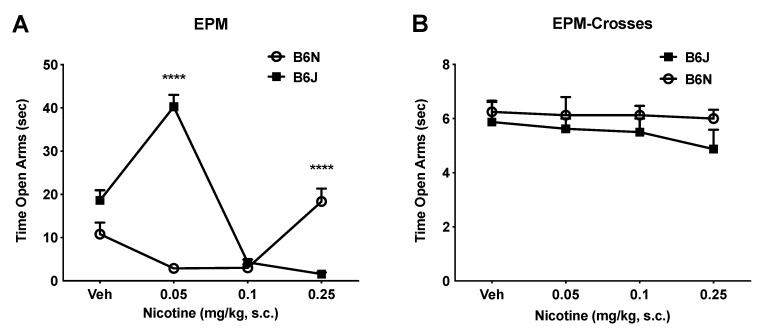
The effects of acute nicotine on anxiety-like behaviors in B6J and B6N substrains. In the B6J strain, mice receiving a lower dose of nicotine (0.05 mg/kg) spent more time in the open arms of the elevated plus maze than mice receiving higher doses (0.1 and 0.25 mg/kg), indicative of anxiolytic-like behavior. In stark contrast, B6N mice show no preference for the open arms of the EPM at 0.05 or 0.1 mg/kg. Only at the highest dose (0.25 mg/kg) do they show a significant increase in the time spent in the open arm of the EPM. There was no effect of nicotine on the total numbers of crosses between arms to suggest non-specific effects of nicotine on locomotion. The data are reported as mean + SE. Each data point is representative of the averaged mouse response at the given dose. Significant difference is based on Holm-Sidak post hoc test following two-way ANOVA between both substrains (**** *p* < 0.0001), *n* = 8/group.

The EPM assay is a well validated anxiolytic drug efficacy test with great predictive validity [34,35]. Similar to a previous report, we demonstrated that nicotine has a dichotomous effect on anxiety-like behavior in the EPM assay [34]. In the B6J strain, we found that mice receiving a lower dose of nicotine (0.05 mg/kg) spent more time in the open arms of the EPM than the mice receiving higher doses (0.1 and 0.25 mg/kg), which is indicative of anxiolytic-like behavior (Figure 2A). This response was observed to have a greater effect on the B6J mice administered the lowest dose of nicotine (0.05 mg/kg), who spent more time in the open arms of the EPM compared to the B6N mice at all administered doses. In contrast, B6N mice showed no preference for the open arms of the EPM at 0.05 or 0.1 mg/kg. Only at the highest dose (0.25 mg/kg) did they show a significant increase in the time spent in the open arm of the EPM. Interestingly, the response for B6J mice was dissipated at this dose, and the B6N mice spent more time in the open arms of the EPM than the B6J mice. The two-way ANOVA of the EPM test revealed significant effects of the dose, strain, and interaction (F_strain_ (1, 56) = 28.27 *p* < 0.0001; F_dose_ (3, 56) = 29.51 *p* < 0.0001; F_strain×dose_ = (3, 56) = 65.27, *p* < 0.0001; Figure 2A). We report no effect of nicotine on the total numbers of crosses between arms, thereby suggesting the non-specific effects of nicotine on locomotion (Figure 2B).

### 3.3. Impact of Repeated Drug Administration on Locomotor Activity

**Figure 3:**
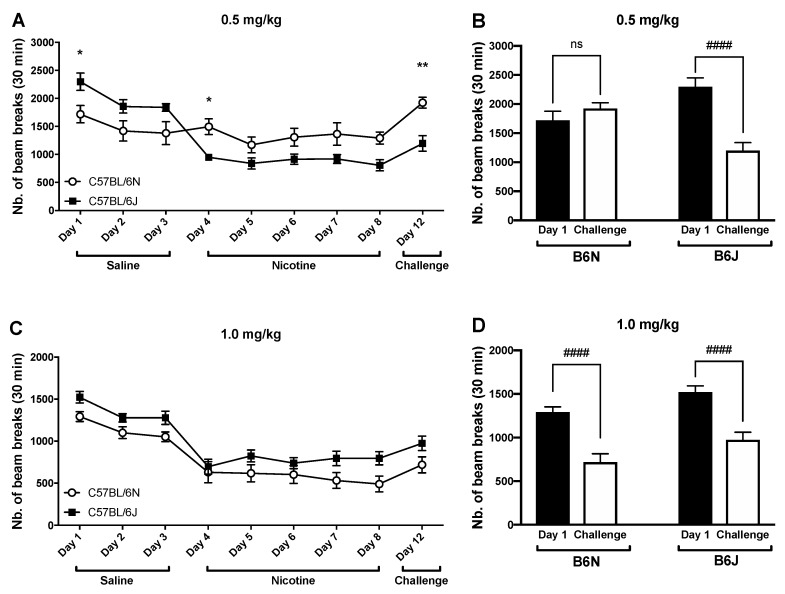
Locomotor sensitization to repeated nicotine administration in male B6J and B6N substrains. Mice were injected s.c. with 0.9% saline for 3 days, nicotine (0.5 or 1.0 mg/kg) for 5 days, and after a washout period (day 6 to day 8), mice were challenged with the same dose of nicotine. Each day, five minutes post subcutaneous administration of the vehicle (0.9% saline) or nicotine, their locomotor activity was measured for 30 min. (**A**,**B**) Locomotor response to 0.5 mg/kg nicotine, and (**C**,**D**) locomotor response to 1.0 mg/kg nicotine. Each data point is representative of the averaged mouse response at the given time-point 30 min post injection. The data are expressed as the number of photocell interruptions and reported as the mean ± SE of 12–15 mice. * *p* < 0.05, ** *p* < 0.01 from Holm-Sidak post hoc test following a multiple t-test analysis between both substrains at various time points, or #### *p* < 0.0001 from the Holm–Sidak post hoc test following a two-way RM repeated measure ANOVA, comparing the averaged mouse response at day 1 and day 12 (challenge day).

At a low dose of nicotine (0.5 mg/kg), differences between the B6J and B6N substrains were observed (Figure 3A). A multiple t-test analysis with Holm–Sidak correction for multiple comparisons shows significant differences between substrains for low-dose nicotine on day 1 (day 1 of saline administration; *p* = 0.02), day 4 (day 1 of nicotine administration; *p* = 0.03), and day 12 (nicotine challenge; *p* = 0.0016). However, mice treated with the first dose of nicotine (day 4) were significantly different from the mice that were challenged with nicotine at day 12 for both strains. When compared to day 1, only B6J mice were significantly different from those challenged with nicotine on day 12 (Figure 3B). At the higher dose of nicotine (1 mg/kg), the multiple t-test analysis with Holm–Sidak correction for multiple comparisons did not yield any significant difference between substrains on any of the days (Figure 3C). Further, when compared to day 1, both the B6J and B6N mice were significantly lower from the respective challenge with nicotine on day 12 (Figure 3D).

### 3.4. Nicotine Metabolism and Kinetics Do not Differ between the B6N and B6J Substrains after Acute Administration of Nicotine

**Figure 4:**
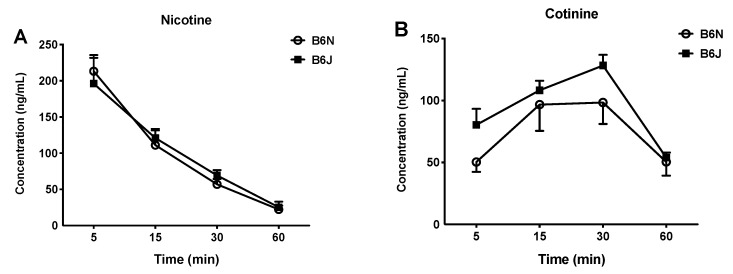
The time course of nicotine and cotinine in male B6J and B6N mice. The rate of nicotine metabolism is not significantly different in the B6J and B6N substrains. Nicotine metabolism was measured by quantifying the production of cotinine after drug administration. Mice were subcutaneously injected with nicotine (1 mg/kg) and had their blood collected at 5, 15, 30, and 60 min after injection. Nicotine levels were measured at 5, 15, 30, 60 min time points. Each time point represents the mean + SEM of six mice per group.

Next, we determined if this effect would be replicated in both substrains demonstrated by differences in the kinetics of nicotine after acute administration. The time course of nicotine and its main metabolite, cotinine, were determined. We observed no significant differences between both strains in the area under the curve (AUC), C_max_, or half-life of the metabolism of nicotine (Figure 4A and Table 2). Interestingly, the pharmacokinetic parameters after acute administration of 1 mg/kg nicotine (s.c.) yielded significant differences in the B6J and B6N mice for the AUC measurements of serum cotinine concentrations. Thus, while both strains showed similar elimination half-lives for nicotine, the overall AUC_0-90_ value of cotinine was significantly higher in the B6J strain (7000 ± 418 min ng/mL) than in the B6N strain (5740 ± 384 min ng/mL), *p* = 0.05 (Figure 4B and Table 2) suggesting differences in cotinine metabolism. The two-way repeated measure RM ANOVA with a Holm–Sidak post hoc test of the nicotine time-course yielded no significant effect of the interaction between time and strain (F_strain×time_ (4,40) = 0.2491, *p* = 0.9085, Figure 4A). It also showed no significant effect for the interaction between time and substrains in the cotinine time-course (F_strain×dose_ (4,40) = 0.5813, *p* = 0.06779, Figure 4B).

### 3.5. B6N and B6J Substrains Differ at Basal Levels

**Figure 5:**
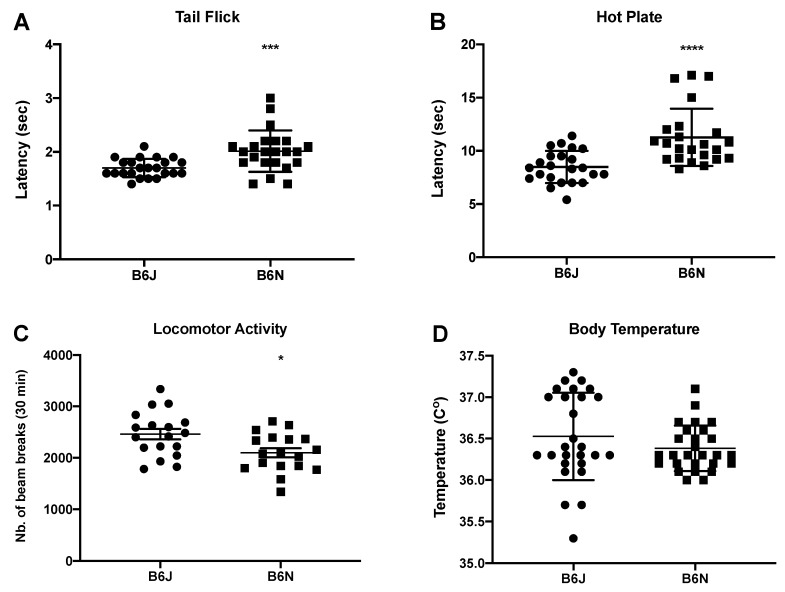
Basal responses for the B6J and B6N substrains. Basal thermal nociception in the male B6J versus B6N substrains demonstrated by latency (sec) in (**A**) the tail flick and (**B**) the hot plate tests (*n* = 23/substrain). (**C**) The basal locomotion response in male B6J and B6N mice (*n* = 18/substrain). (**D**) The basal body temperature in male B6J and B6N mouse strains (*n* = 27/substrain). All data are shown as the mean ± SE. Each data point is representative of each individual mouse response. Significant differences between substrains are based on an unpaired t-test (* *p* < 0.05, *** *p* < 0.001, **** *p* < 0.0001).

Baseline responses in B6N and B6J mice in the tail-flick, hot plate, locomotion test, and body temperature from the previous experiments were analyzed. Figure 5 demonstrates that the B6N and B6J substrains differ at basal levels. Significant differences were observed in the tail-flick (*t* = 3.563, df = 44, *p* = 0.0009), hot plate (*t* = 4.324, df = 44, *p* < 0.0001), and locomotor activity (*t* = 2.680, df = 34, *p* = 0.0113) tests based on an unpaired two-sided *t*-test as seen in Figure 5A–C, respectively. Although the B6N mice displayed higher locomotion and nociceptive latencies in the tail flick and hot plate assay (indicative of enhanced nociceptive thresholds compared to the B6J mice), no significant differences were observed in the body temperatures between both substrains (*t* = 1.261, df = 52, *p* = 0.2130), as shown in Figure 5D.

## 4. Discussion

The primary goal of this paper was to evaluate the differences between the C57BL/6N (B6N) and C57BL/6J (B6J) mouse substrains in response to acute and chronic nicotine. We report, for the first time, substrain differences in the pharmacological effects of acute and repeated nicotine administration in these substrains. We expanded the catalog of behavioral differences in the sensitivity between the B6J and B6N substrains in several pharmacological measures: locomotion (after acute and repeated exposure), body temperature, nociception, and anxiety-like behaviors. Overall, we identified enhanced nicotine sensitivity to antinociception, hypothermia, locomotion, and anxiety-like behaviors in the B6J versus B6N substrains after acute dosing (Figure 1). Here, we find that nicotine produced a dose-dependent increase in latency in the tail-flick and hot plate tests in both the B6J and B6N substrains (Figure 1A,B). However, the B6J substrain demonstrates a more pronounced thermal antinociceptive response in both the tail-flick and hot plate test, as well as an increased potency to nicotine in both tests compared to B6J mice (Table 1). In addition, nicotine dose-dependently induced hypothermia in both the B6J and B6N substrains (Figure 1D). Again, the B6J substrain demonstrated a more pronounced response to nicotine-induced hypothermia with an increased potency to nicotine compared to the B6N strain (Table 1). 

Locomotor activity has long been widely used to study the effects of nicotine in rodents [22,36,37,38]. Nicotine produced a reduction in locomotion in a dose-related manner in both substrains. This effect is more pronounced in the B6J substrain, demonstrating an increased sensitivity to nicotine compared to the B6N mice (Figure 1C). We then explored the difference in locomotor sensitization between the two substrains after repeated administration of nicotine. Literature has demonstrated that upon repeated administration of psychostimulants, animals display a progressively greater behavioral response, regulated by “experience-dependent neuronal plasticity”, which is thought to be a key event in addiction [22,39]. However, our results did not show a sensitized locomotor response in either of the substrains after repeated exposure to nicotine at the two doses tested (0.5 and 1 mg/kg). Nevertheless, tolerance to the locomotor depressant effect of a challenge dose of nicotine on day 12 was observed after repeated injection of nicotine at 0.5 mg/kg in the B6N mice only (Figure 3). At the higher dose of nicotine (1 mg/kg), both substrains did not display any significant sensitization after repeated administration of the drug. In contrast to nicotine, the B6J mice showed a larger sensitized locomotor response to cocaine than the B6N mice, as reported by Kumar et al., 2013 [22]. This difference of sensitivity in locomotor activity within the closely related B6N and B6J substrains has helped identify a nucleotide level mutation that regulates the cocaine response within the Cyfip2 using QTL analysis. In the EPM assay, we demonstrated that nicotine produces a dichotomous effect on anxiety-like behaviors. In B6J mice, only the lower doses of nicotine (0.05 mg/kg) increased the time spent in the open arms of the EPM compared to the vehicle group, which is indicative of anxiolytic-like behavior (Figure 2A). This effect dissipated at higher doses (0.1 and 0.25 mg/kg). In contrast, an increase in the time spent in the open arms was only seen at the highest dose of nicotine (0.25 mg/kg) in the B6N mice. Overall, our findings are consistent with previously published mouse studies that show that low doses of nicotine promote anxiolytic-like behavior, while higher doses promote anxiogenic-like behaviors [34,40]. This is clinically relevant to human smokers because studies reveal that many smokers report tobacco-use as a way to relieve anxiety. Importantly, the effect of nicotine on anxiety in humans appears to multifactorial, including a genetic predisposition for anxiety. Lasser et al. report that individuals with a diagnosis of anxiety disorders are twice as likely to smoke than other persons [41].

Next, we explored if pharmacokinetic differences within the substrains could explain the observable phenotypic differences by measuring the time course of nicotine (Figure 4A) and its main active metabolite, cotinine (Figure 4B). We found that these differences are not due to differences in nicotine pharmacokinetics (Table 1). The AUC, C_max_, and elimination half-life of nicotine were similar in both substrains and consistent with published studies on nicotine pharmacokinetics in C57BL/6J mice [42]. However, while both strains showed a similar elimination of half-live for nicotine, the cotinine levels were significantly higher in B6J mice (Table 2). While it is possible that differences in cotinine pharmacokinetics could have potentially contributed to the observed substrain pharmacological differences, we believe that this contribution is minimal, since cotinine is reported to be inactive after systemic administration in mice in the pharmacological responses measured in our studies [43]. Interestingly, C57BL/6J mice have been shown to possess a higher clearance rate of cotinine compared to DBA/2J mice in vivo, despite having similar nicotine clearance [42]. Finally, differences in basal levels of nociceptive responses in the tail-flick and hot-plate tests, as well as differences in locomotor activity counts were found between the B6J and B6N mice. The B6N substrain baseline nociceptive latencies were significantly higher than those of the B6J mice in both tests. However, their baseline locomotor counts were significantly lower than those of the B6J mice. These findings are similar to those previously reported in these two substrains [20,22,44]. In addition, no significant differences were found in the basal body temperature between the two substrains.

The heritable differences in the basal and nicotine response differences described here lend themselves to genetic mapping using a RCC strategy. Such a strategy has been successful in QTLs for behavior, immunity, and physiological phenotypes [20,21,22,45,46]. This approach has most widely been utilized in C57BL/6 substrains and has recently been used in non-obese diabetic (NOD) substrains [47]. Presumably, with only 34 SNPs, 2 Indels, and 15 structural variants that lead to coding changes, causative changes can be quickly identified. It is highly likely that many of these phenotypic changes are due to eQTLs that are caused by variants in enhancers and promoters. There are an estimated 10,794 total variants between B6J and B6N, or roughly one variant every 260 kb. Given a typical QTL interval of 20 Mb from a F2 cross, there are expected to be 77 variants between B6N and B6J. The causal variant can be further filtered with gene expression differences in the tissue of interest. The similar size interval between a DBA/2J and B6J cross would contain 31914 variants [18]. Thus, the task of identifying causal variants is highly simplified in the RCC by several orders of magnitude. We also predict that any variant would have pleiotropic effects. A single variant may be responsible for many for of the observed basal and nicotine induced changes. A mapping QTL analysis for the basal and nicotine-induced phenotypes will allow us to better determine the relationship between these phenotypes. For example, we expect genes that modify nicotine responses to potentially modify the basal locomotor changes that we observed.

## 5. Conclusions

In conclusion, our present findings suggest that B6 mouse substrains may be useful for genetic studies to elucidate some of the genetic differences involved in nicotinic behaviors. This is important to note because one’s initial sensitivity to a drug plays a critical role in their vulnerability to dependence and addiction [48]. Since most cigarette smokers do so to relieve the stress, anxiety, and withdrawal symptoms that come with nicotine dependence, the anxiolytic-like effects of nicotine are also essential to study. A limitation of our study is the use of only male animals. We acknowledge that it would be interesting to see if these same substrain differences exist within female cohorts. Another potential limitation is the use of vendor purchased mice rather than in-house bred mice, which could have contributed to the observed behavioral differences. However, a previously published study found that mice bred in-house versus mice purchased from a vendor did not show significant alterations in their behavior in several measures, including anxiety-like testing and locomotor activity, provided that the mice were sufficiently acclimated upon arrival [49]. In our hands, mice were acclimated to the vivarium for a week and handled before any behavioral experimentation. In addition, another study reported that vendor breeding versus in-house breeding did not alter the B6 substrain differences in alcohol preference and consumption [50]. While the risk of random epigenetic changes occurring from vendor breeding is probable, the studies mentioned above support the hypothesis that B6 substrain differences are most likely due to genetic differences rather than environmental differences. Future studies will aim to test whether the Cyfip2 (S968F) variant that is known to regulate psychostimulant response could also contribute to the differences seen in nicotine response.

## Figures and Tables

**Table 1:** A summary of nicotine’s potency in the tail-flick, hot plate, body temperature and locomotor activity tests after acute administration in male B6J and B6N mice. Potency is expressed as the ED50 ± confidence limits (mg/kg). Each group contained 8 mice. * CL of ED50 values do not overlap.

Test	B6J (mg/kg)	B6N (mg/kg)
**Tail flick**	1.3 (0.56–1.89)	5.9 (3.1–11.23) *
**Hot plate**	1.08 (0.83–1.38)	7.79 (3.56–13.41) *
**Body temperature**	0.57 (0.37–0.88)	1.15 (0.96–2.13) *

**Table 2:** Summary of pharmacokinetic parameters after acute nicotine administration (1 mg/kg, s.c.) in male B6J and B6N mice. Statistical comparisons were performed using two-tailed t-test. N.S = Not significant.

	Nicotine				Cotinine		
Mean (SE)	AUC_0-90_ (min ng/mL)	AUC_0-inf_ (min ng/mL)	C_max_ (ng/mL)	T_1/2_ (min)	AUC_0-90_ (min ng/mL)	AUC_0-inf_ (min ng/mL)	C_max_ (ng/mL)
6N	4610 (126)	5200 (136)	216 (20.6)	17.2 (1.43)	**5740** (384)	7230 (713)	129 (12.9)
6J	4930 (380)	5880 (741)	206 (30)	20.5 3.68	**7000** (418)	8780 (506)	131 (7.16)
Comparison	N.S.	N.S.	N.S.	N.S.	P = 0.05	N.S.	N.S.
